# Efficacy and safety of mirogabalin for chemotherapy-induced peripheral neuropathy: a prospective single-arm trial (MiroCIP study)

**DOI:** 10.1186/s12885-023-11560-4

**Published:** 2023-11-11

**Authors:** Sonoko Misawa, Tadamichi Denda, Sho Kodama, Takuji Suzuki, Yoichi Naito, Takahiro Kogawa, Mamoru Takada, Tomoki Suichi, Kazuhito Shiosakai, Satoshi Kuwabara, on behalf of the MiroCIP study group, Go Saito, Aoi Hino, Shunsuke Imanishi, Norio Ureshino, Daisuke Satomi, Yuko Tanabe, Yutaka Hanaoka, Atsushi Miyamoto, Takeshi Suzuki, Atsushi Naganuma, Yasuhiro Yanagita, Katsutoshi Sekine, Fumihiko Kusano, Masato Nakamura, Hiroshi Imazeki

**Affiliations:** 1 Department of Neurology, Graduate School of Medicine, Chiba University, 1-8-1 Inohana, Chuo-Ku, Chiba-Shi, Chiba, 260-8677 Japan; 2 Division of Gastroenterology, Chiba Cancer Center, 666-2 Nitona-Cho, Chuo-Ku, Chiba-Shi, Chiba, 260-8717 Japan; 3 Primary Medical Science Department, Medical Affairs Division, Daiichi Sankyo Co., Ltd, 3-5-1 Nihonbashi Honcho, Chuo-Ku, Tokyo, 103-8426 Japan; 4 Department of Respirology, Graduate School of Medicine, Chiba University, 1-8-1 Inohana, Chuo-Ku, Chiba-Shi, Chiba, 260-8670 Japan; 5 Department of General Internal Medicine/Experimental Therapeutics/Medical Oncology, National Cancer Center Hospital East, 6-5-1 Kashiwanoha, Kashiwa-Shi, Chiba, 277-8577 Japan; 6 Division of Early Clinical Development for Cancer, Department of Advanced Medical Development, Cancer Institute Hospital of JFCR, 3-8-31 Ariake, Koto-Ku, Tokyo, 135-8550 Japan; 7 Department of General Surgery, Graduate School of Medicine, Chiba University, 1-8-1 Inohana, Chuo-Ku, Chiba-Shi, Chiba, 260-8670 Japan; 8 Data Intelligence Department, Global DX, Daiichi Sankyo Co., Ltd, 1-2-58 Hiromachi, Shinagawa-Ku, Tokyo, 140-8710 Japan

**Keywords:** Analgesia, Cancer, Chemotherapy, Chemotherapy-induced peripheral neuropathy, Mirogabalin, Neurotoxicity, Oxaliplatin, Pain, Taxane

## Abstract

**Background:**

Chemotherapy-induced peripheral neuropathy (CIPN) is a painful, dose-limiting adverse effect of commonly used chemotherapeutic agents. The purpose of this exploratory study was to evaluate the efficacy and safety of mirogabalin in patients with moderate to severe CIPN during chemotherapy and the effects of 12 weeks’ intervention on chemotherapy completion and CIPN severity.

**Methods:**

Patients experiencing moderate to severe CIPN while undergoing oxaliplatin- or taxane-containing chemotherapy for colorectal, gastric, non-small-cell lung, or breast cancer received mirogabalin at between 5 and 15 mg twice daily. The primary endpoint was change in numeric rating scale (NRS) score for pain from baseline to week 12. Secondary endpoints included NRS scores for tingling and sleep, completion of chemotherapy, severity of CIPN, and quality of life (QOL) scores. The safety endpoint was incidence of adverse events.

**Results:**

Of 58 patients who consented to participation, 52 were eligible and constituted the full analysis set and safety analysis set. From baseline to week 12 (last observation carried forward [LOCF]), NRS score decreased by 30.9%: mean change (95% confidence interval [CI]), − 1.7 (− 2.4 to − 1.0) (*p* < 0.001). Patients with baseline NRS of ≥ 6 experienced a 44.0% reduction in score from baseline to week 12 (LOCF): mean change (95% CI), − 3.3 (− 5.0 to − 1.5) (*p* = 0.002). Chemotherapy was discontinued in 18 (34.6%) patients; CIPN led to discontinuation in only 2 (3.8%). There was no notable worsening of CIPN severity in terms of Common Terminology Criteria for Adverse Events grade or Modified Total Neuropathy Score-reduced, although use of pain medications during chemotherapy might cause worsening of CIPN due to underestimation of subjective symptoms. QOL score based on the EuroQol five-dimensional descriptive system did not worsen during the 12 weeks. Thirty-one percent of patients experienced adverse drug reactions, and the most common event was somnolence (13.5%). Serious adverse events and death occurred in 3 patients and 1 patient, respectively; however, they were unrelated to mirogabalin treatment.

**Conclusions:**

Intervention with mirogabalin during chemotherapy may be effective and safe for cancer patients with moderate to severe CIPN. It can contribute to completion of chemotherapy without worsening of CIPN.

**Trial registration:**

Japan Registry of Clinical Trials (jRCTs031210101, registered 20/5/2021).

**Supplementary Information:**

The online version contains supplementary material available at 10.1186/s12885-023-11560-4.

## Background

Chemotherapy-induced peripheral neuropathy (CIPN) is a major adverse event affecting patients receiving treatment with neurotoxic chemotherapeutic agents including platinum, taxanes, and vinca alkaloids [1]. The prevalence of CIPN has been reported in the range of 12% to 96% [2]. Because CIPN causes pain and tingling in the extremities, patients often need neuropathic pain medication.

Many clinical trials relating to pain treatment of CIPN have been reported. Most of these previous studies were conducted in patients with chronic CIPN after completion of chemotherapy, and only a few studies evaluated patients with CIPN during or immediately after chemotherapy. Therefore, although the management of pain caused by CIPN during the course of chemotherapy is a common problem in real-world clinical practice, there is no consensus about the management of CIPN during chemotherapy. In daily practice, chemotherapy is reduced or discontinued based on the patient’s subjective pain intensity, resulting in negative consequences for patient outcomes [2, 3].

When neuropathic pain medications are used during chemotherapy, the severity of CIPN specifically should be appropriately assessed in parallel with the assessment of neuropathic pain, according to the American Society of Clinical Oncology (ASCO) guideline [4]. Subjective assessment of pain relief by patients receiving pain medication may result in underestimation of the progression of CIPN and lead to more severe sequelae by missing the optimal time to discontinue chemotherapy. Futhermore, it can significantly reduce the patient’s quality of life (QOL) after the end of cancer treatment.

Several treatment options are currently available for CIPN, including selective serotonin–norepinephrine reuptake inhibitors (e.g. duloxetine), gabapentinoids (pregabalin, gabapentin), and tricyclic antidepressants (e.g. amitriptyline) [5–12]. However, ASCO only moderately or weakly recommends duloxetine, and pregabalin is not recommended in the guideline [4]. Therefore, new treatment options are needed for CIPN.

Mirogabalin besilate (henceforth referred to as mirogabalin) is an oral gabapentinoid drug with analgesic effects resulting from its ability to bind to the α_2_δ subunit of voltage-gated calcium channels [13]. Mirogabalin has been approved in several Asian countries for the treatment of neuropathic pain including both peripheral and central neuropathic pain [14, 15]. The results of phase 3 clinical trials and a meta-analysis support its efficacy against neuropathic pain [16–20]. However, clinical evidence for the efficacy and safety of mirogabalin in patients with CIPN is limited [21, 22].

In the present study, from the perspective of real-world medical practice, we evaluated the efficacy of mirogabalin for the treatment of pain in patients who developed moderate to severe CIPN during chemotherapy with oxaliplatin or taxane for four types of solid cancers (colorectal, gastric, non-small-cell lung, and breast cancers). We also examined its influence on the completion of chemotherapy and the risk of worsening of CIPN due to underestimation of pain under mirogabalin treatment.

## Methods

### Study design

**Fig. 1:**
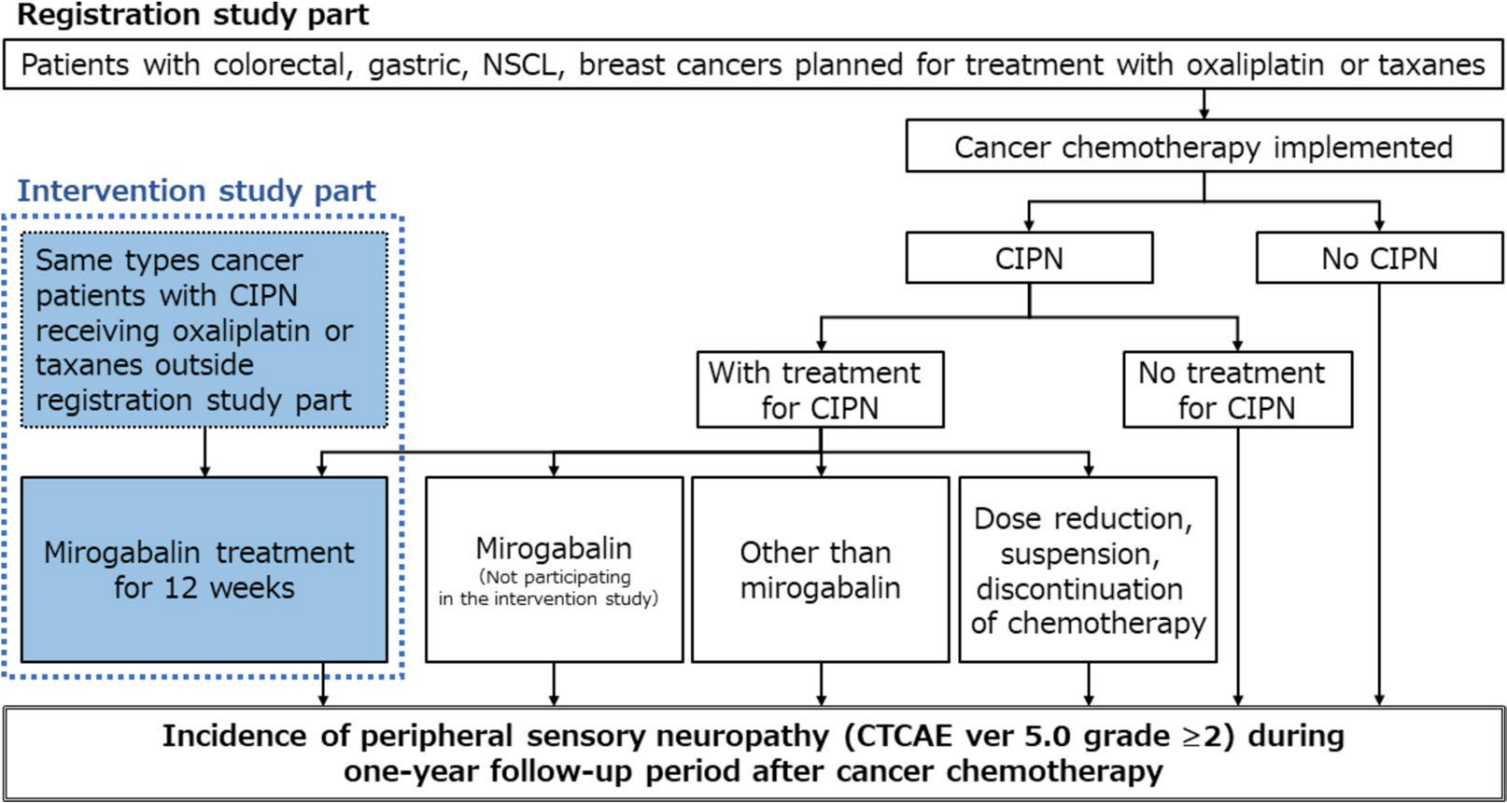
Design of the MiroCIP study, which comprised a registrational study and an interventional study. Same-type cancer patients with chemotherapy-induced peripheral neuropathy (CIPN) and receiving oxaliplatin or taxanes outside the registration study part did not participate in the 1-year follow-up registration part of the MiroCIP study. *CTCAE*, Common Terminology Criteria for Adverse Events; *NSCL*, non-small-cell lung

The present study was conducted as part of the MiroCIP study, carried out in Japan. MiroCIP study comprised two parts: a multicenter prospective ongoing registrational study that started in May 2021, and this exploratory, interventional, open-label, single-arm study carried out between May 2021 and September 2022 (Fig. 1). The aim of the registrational study is to investigate the incidence, risk factors, clinical features, and prognosis of CIPN in patients receiving oxaliplatin- or taxane-containing chemotherapy, whereas the aim of this interventional study was to investigate the efficacy and safety of mirogabalin in patients with CIPN.

### Trial registration

The MiroCIP study is registered in the Japan Registry of Clinical Trials under the identifier jRCTs031210101 (date of registration, 20/5/2021).

### Participating institutions

The present interventional part of MiroCIP study was carried out at 12 institutions across Japan (Supplementary Table 1).

### Patients

Key eligibility criteria included age ≥ 20 years, a diagnosis of CIPN grade ≥ 2 according to the Common Terminology Criteria for Adverse Events (CTCAE) version 5.0, and a pain numerical rating scale (NRS) score ≥ 4; patients had colorectal, gastric, non-small-cell lung, or breast cancer and were undergoing chemotherapy with a regimen including oxaliplatin or taxane. Patients with pain due to causes other than CIPN (as carefully judged by the attending physician), with allergy to mirogabalin, and with major organ complications were excluded. Full eligibility criteria are provided in the Supplementary Methods.

Of the patients enrolled in the registrational study or from outside the registrational study, those who provided written informed consent to participate in and met the eligibility criteria for this interventional study were enrolled. Most patients were enrolled from the MiroCIP registrational study, but 16 patients were enrolled from outside the MiroCIP registrational study to ensure the pre-specified target sample size.

### Treatment

The treatment period was 12 weeks. Mirogabalin was administered orally at 5 mg twice daily during the first week of the titration period and was titrated up to 10–15 mg twice daily in the following weeks based on age, symptoms, and renal function. For patients with moderate renal impairment (i.e. creatinine clearance 30 to < 60 mL/min), the maintenance dose was 5–7.5 mg twice daily according to the package insert [14].

Prohibited medications included pregabalin, gabapentin, carbamazepine, duloxetine, and lorazepam. The use of compression therapy, cryotherapy, or acupuncture was also prohibited. Co-administration of opioids, non-steroidal anti-inflammatory drugs (NSAIDs), and acetaminophen was permitted if they were administered prior to enrollment and no changes in dosage or administration were made during the study period.

### Endpoints

The primary efficacy endpoint was the change in NRS pain score from baseline to week 12 [23]. Patients were asked to rate the pain they had experienced over the previous 7 days on an 11-point NRS ranging from 0 (“no pain”) to 10 (“worst pain possible”).

Secondary efficacy endpoints included changes from baseline to weeks 4 and 12 in NRS scores in the last 7 days for tingling (0 = “no tingling” to 10 = “worst tingling possible”) and sleep disturbance (0 = “no sleep disturbance” to 10 = “sleep completely disturbed by pain”); cases of dose reduction, suspension, and discontinuation of chemotherapy during the study period; sensory CIPN severity as assessed by CTCAE version 5.0 at baseline, week 4, and week 12, the Functional Assessment of Cancer Therapy/Gynecologic Oncology Group Neurotoxicity subscale (FACT/GOG-NTX) [24] at baseline, week 4, and week 12, and Modified Total Neuropathy Score-Reduced (TNSr) [25] at baseline and week 12; and QOL as assessed by the EuroQol five-dimensional descriptive system (EQ-5D-5L version) [26] and Patient Global Impression of Change (PGIC) scale (1 = “very much improved” to 7 = “very much worse” since the start of treatment) [27] at week 12.

The permissions for the FACT/GOG-NTX and Modified TNSr were obtained in advance as follows. For FACT/GOG-NTX, the FACIT and all related works are owned and copyrighted by and the intellectual property of David Cella, Ph.D; permission for use of the FACT/GOG-NTX is obtained by contacting Dr. Cella at information@facit.org. For the Modified TNSr, all licensed works and derivative works shall be marked as appropriate with the following: “Copyright Johns Hopkins University (2023). All rights reserved.”

The safety endpoint was the incidence of adverse events (AEs). AEs were coded using the Japanese version of the Medical Dictionary for Regulatory Activities, version 25.0.

### Sample size

The target sample size was set at 55 patients for feasibility reasons, as this was an exploratory interventional study. In previous clinical trials on the efficacy of mirogabalin, the standard deviations (SDs) for the change in NRS score from baseline to 12 weeks were 1.5 [16] and 2.0 [17]. Assuming SDs of 2.0 and 3.0 for change of NRS score and a 10% dropout rate, the distance from the mean to the limit of the 95% confidence interval (CI) (one-sided) for 50 subjects was 0.55–0.83.

### Statistical analyses

Efficacy analyses were carried out using data for the full analysis set (FAS), defined as all eligible patients who had received at least one dose of the study drug and for whom baseline data were available. Supplementary analyses for efficacy endpoints were conducted using data for the per protocol set (PPS), defined as all patients in the FAS whose treatment was provided in compliance with the study protocol and the Japanese package insert of mirogabalin [14]. The safety analysis used data for the safety analysis set, defined as all patients who had received at least one dose of the study drug.

For the primary endpoint, the primary analysis was performed by calculating summary statistics, 95% CIs and *p* values vs baseline, using paired *t*-tests. The last observation carried forward (LOCF) method was applied to supplement missing data of NRS scores for pain at week 12. Under this method, any data missing at 12 weeks were imputed as the data recorded at the latest observation since the start of mirogablin treatment, including data recorded at the visit at which treatment was discontinued.

Statistical analysis was carried out using SAS version 9.4 (SAS Institute Inc., Cary, NC, USA). The significance level for hypothesis testing was set at 5% (two-sided), and the CI for both sides was 95%. Because the study was exploratory, no adjustments were made for multiple comparisons.

## Results

### Patients

**Fig. 2:**
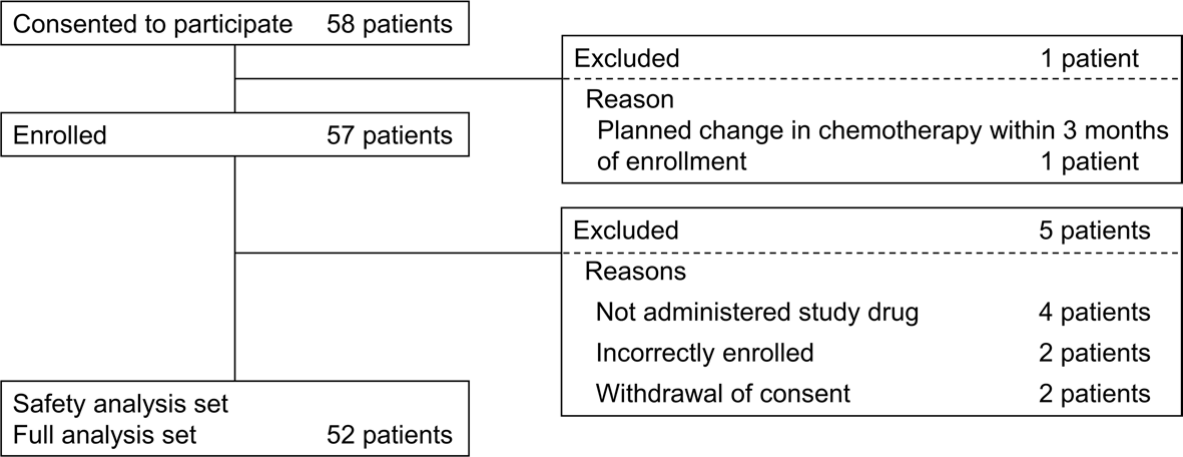
Patient disposition. The per protocol set (PPS) comprised 30 patients, after exclusion of 22 patients from the full analysis set (FAS) for violations of protocol (study drug not administered in compliance with the Japanese package insert of mirogabalin, such as not at twice-daily dosage and not at an effective dosage, *n* = 20; other violations of eligibility criteria or protocol, *n* = 2). A total of 40 patients in the FAS and 24 patients in the PPS completed the study; most withdrawals were at the patients’ request

Fifty-eight patients were screened, 57 were enrolled, and 52 received at least one dose of study drug (FAS) (Fig. 2). After exclusion of 2 patients with protocol violations and 20 patients whose use of mirogabalin did not follow the instructions on the Japanese package insert, 30 patients constituted the PPS.

Patient baseline characteristics are summarized in Table 1 and Supplementary Table 2. The commonest cancer was colorectal cancer (65.4%), followed by non-small-cell lung, breast, and gastric cancer (13.5%, 11.5%, and 9.6%, respectively). Almost half of patients (48.1%) had stage IV cancer. All patients had good performance status (performance status 0, 69.2%; 1, 30.8%), and 86.5% of patients were undergoing chemotherapy in the non-perioperative period. Chemotherapy included oxaliplatin in about two-thirds of cases (65.4%), and taxane in the remainder (34.6%). Almost one third of patients had experienced neurotoxic chemotherapy (17/52, 32.7%). Similar baseline characteristics were observed in the PPS (Supplementary Table 3).

**Table 1:** Baseline demographic and clinical characteristics of patients with chemotherapy-induced peripheral neuropathy in the MiroCIP interventional study (full analysis set)

Characteristic	Full analysis set (*n* = 52)^a^
Age, years	65.5 ± 9.9
Sex, male/female	29 (55.8) / 23 (44.2)
Body mass index, kg/m^2^	22.50 ± 3.25
Creatinine clearance, mL/min	83.85 ± 26.57
Smoking status, current/previous/never	4 (7.7) / 26 (50.0) / 22 (42.3)
Alcohol consumption habit	34 (65.4)
Cancer type, C / G / N / B	34 (65.4) / 5 (9.6) / 7 (13.5) / 6 (11.5)
Cancer stage, II / III / IV / R	1 (1.9) / 6 (11.5) / 25 (48.1) / 20 (38.5)
Performance status, 0/1	36 (69.2) / 16 (30.8)
Timing of chemotherapy, post/non-peri	7 (13.5) / 45 (86.5)
History of radiotherapy	6 (11.5)
Chemotherapeutic agent included in regimen, O/T	34 (65.4) / 18 (34.6)
Accumulated dose per body surface area, mg/m^2^, O/T^b^	1339.52 ± 710.17 / 1310.49 ± 1668.97
History of neurotoxic chemotherapy	17 (32.7)
Oxaliplatin	11 (21.2)
Carboplatin	3 (5.8)
Cisplatin	2 (3.8)
Docetaxel	2 (3.8)
Paclitaxel	2 (3.8)

*B* Breast cancer; *C* Colorectal cancer; *FAS* Full analysis set; *G* Gastric cancer *non-peri* Non-perioperative; *N* Non-small-cell lung cancer; *O* Oxaliplatin; *Post* Postoperative; *R* Recurrence after surgery; *SD* Standard deviation; *T* Taxane

^a^Mean ± SD or *n* (%)

^b^These data were at the end of the study

### Changes in NRS score for pain

**Fig. 3:**
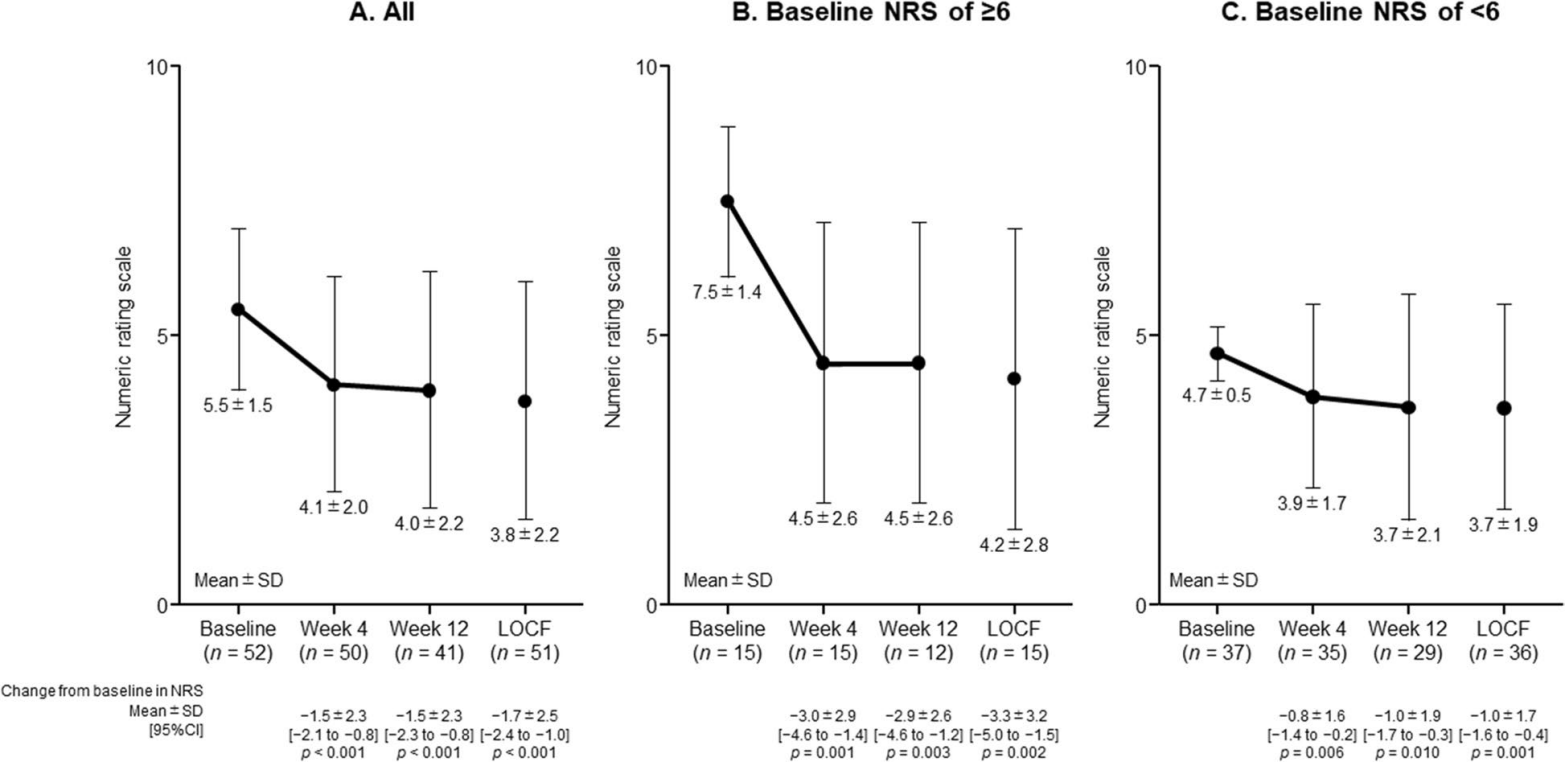
Pain (including tingling) assessed by numeric rating scale score over 12 weeks of mirogabalin treatment: in the total (full analysis set) (**A**) and in subgroups with baseline NRS score ≥ 6 (**B**) or < 6 (**C**). Data are presented as mean ± SD [95% CI]. The *p* values, determined by paired *t*-test, are vs baseline. *CI*, confidence interval; *LOCF*, last observation carried forward; *NRS*, numeric rating scale; *SD*, standard deviation

Results for the primary efficacy endpoint (Fig. 3A) showed a significant decrease in the NRS pain score from baseline to week 12, with a mean change [95% CI] of − 1.5 [− 2.3 to − 0.8] (*p* < 0.001), and similar results were obtained when using LOCF data (− 1.7 [− 2.4 to − 1.0], *p* < 0.001; − 30.9 reduction from baseline). The most significant reduction occurred during the first 4 weeks of treatment, and this reduction was maintained at week 12. A greater reduction was also achieved in patients with baseline NRS ≥ 6 than in those with baseline NRS < 6 (Fig. 3B, C). The trend of reduction in NRS scores (mean, 95% CI) was also seen in patients receiving either oxaliplatin (*n* = 29, mean change [95% CI], − 1.8 [− 2.6 to − 0.9]; *p* < 0.001) or taxane (*n* = 12, − 0.9 [− 2.4 to 0.6]; *p* = 0.204) (data not shown).

The trend in reduction in NRS score from baseline was not clinically meaningful different irrespective to presence or absence of dose reduction, suspension, or discontinuation of chemotherapy due to all causes. The change in NRS score from baseline to week 12 was − 1.4 [− 2.3 to − 0.6]; *p* = 0.002 (*n* = 26) and − 1.7 [− 3.1 to − 0.3]; *p* = 0.020 (*n* = 15) in patients with and without dose reduction, suspension, or discontinuation of chemotherapy due to all causes, respectively (data not shown).

Similar results were obtained using PPS data (mean change [95% CI], − 1.5 [− 2.2 to − 0.8]; *p* < 0.001) (Supplementary Table 4).

### Changes in NRS scores for tingling and sleep disturbance

Supplementary Table 4 summarizes the NRS data for tingling and sleep disturbance. NRS score for tingling decreased significantly from baseline to week 12, showing a mean change [95% CI] of − 1.2 [− 1.9 to − 0.4] (*p* = 0.003). Mean [95% CI] change in NRS score for sleep disturbance from baseline to week 12 was − 0.2 [− 0.8 to 0.4] (*p* = 0.534). Equivalent results were obtained for the PPS.

### Dose reduction, suspension, or discontinuation of chemotherapy due to CIPN

The incidences of chemotherapy dose reduction, suspension, and discontinuation due to all causes were 7.7%, 23.1%, and 34.6%, and those due to CIPN were 5.8%, 1.9%, and 3.8%, respectively (Table 2). Similar results were obtained for the PPS (Supplementary Table 5).

**Table 2:** Incidence of dose reduction, suspension, or discontinuation of chemotherapy^a^ in patients with chemotherapy-induced peripheral neuropathy (full analysis set, *n* = 52)

Event	*n* (%)
**Due to all causes**	
No dose reduction, suspension, or discontinuation	20 (38.5)
Dose reduction	4 (7.7)
Suspension	12 (23.1)
Discontinuation	18 (34.6)
**Due to CIPN**	
No dose reduction, suspension, or discontinuation	46 (88.5)
Dose reduction	3 (5.8)
Suspension	1 (1.9)
Discontinuation	2 (3.8)

*CIPN,* Chemotherapy-induced peripheral neuropathy

^a^Including oxaliplatin or a taxane

### Changes in CIPN severity

**Fig. 4:**
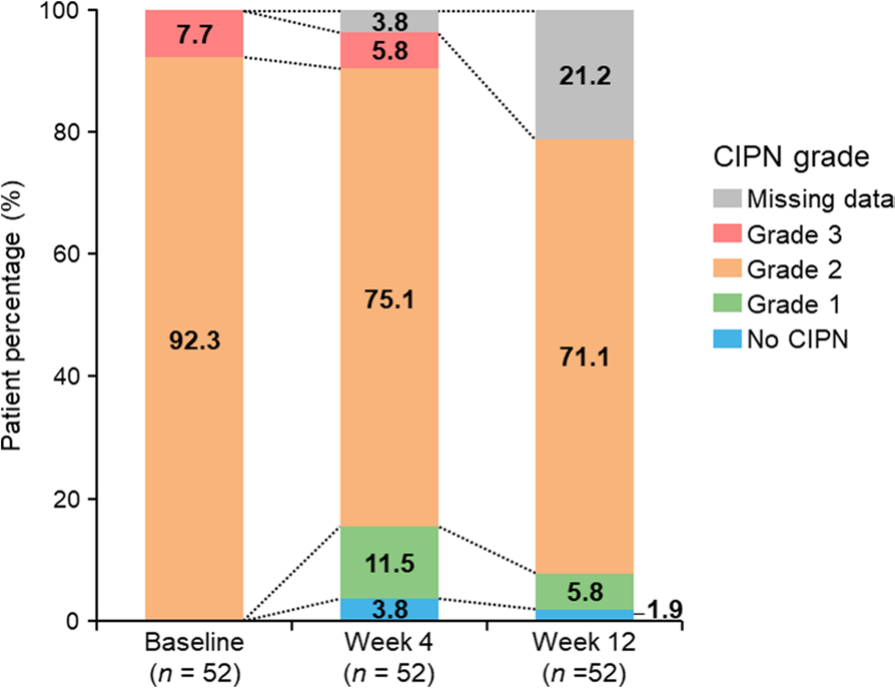
Numbers of patients with change in grade of chemotherapy-induced peripheral neuropathy according to the Common Terminology Criteria for Adverse Events over 12 weeks (full analysis set). Data are presented as percentage of patients. *CIPN*, chemotherapy-induced peripheral neuropathy

At baseline, 92.3% (48/52) of patients had grade 2 CIPN (Fig. 4). The grade remained unchanged in most patients, and some patients showed improvement at weeks 4 and 12 (Supplementary Table 6).

Changes in FACT/GOG-NTX and Modified TNSr total scores for the FAS and PPS are summarized in Supplementary Table 7. The total FACT/GOG-NTX score (mean ± SD) was 12.7 ± 6.9 at baseline and tended to improve to 10.8 ± 7.4 at week 12 (*p* = 0.114). The mean change (95% CI) in Modified TNSr total score from baseline to week 12 was − 0.5 (− 1.5 to 0.5) (*p* = 0.284). An increased score of Modified TNSr indicates worsening of neuropathy.　Similar results were observed for CTCAE grade of CIPN, FACT/GOG-NTX, and Modified TNSr total score in the PPS.

### Changes in EQ-5D-5L index value and PGIC score

The change in EQ-5D-5L index value from baseline to week 12 was 0.0128 (95% CI, − 0.0406 to 0.0663) (*p* = 0.630) (Supplementary Table 8). Regarding PGIC scores at week 12, most patients with available data had at least minimal improvement from baseline; 24.2% (8/33) had scores of 2 (at least “much improved”) and 72.7% (24/33) had scores of 3 (at least “minimally improved”). Similar results were found for the PPS.

### Safety

The incidence of treatment-emergent adverse events (TEAEs) was 76.9% (40/52 patients), and the incidence of AEs related to the study drug (ADRs) was 30.8% (16/52 patients) (Table 3). The most common ADRs were somnolence (13.5%), dizziness (9.6%), and oedema peripheral (3.8%). The ADRs leading to discontinuation were dizziness (*n* = 2), somnolence (*n* = 1), oedema peripheral (*n* = 1), abdominal discomfort (*n* = 1), altered state of consciousness (*n* = 1), and hepatic function abnormal (*n* = 1). One death occurred due to COVID-19, and it was not related to mirogabalin. Serious AEs other than death occurred in three patients (one with pyelonephritis associated with cancer progression, one with progression breast cancer, and one with COVID-19); however, none were related to mirogabalin. All data for TEAEs are summarized in Supplementary Table 9.

**Table 3:** Incidences of adverse drug reactions in patients with chemotherapy-induced peripheral neuropathy in the MiroCIP interventional study (safety analysis set)

ADR^a^	*n* (%)
Overall	16 (30.8)
Occurring in ≥ 1 patient	
Somnolence	7 (13.5)
Dizziness	5 (9.6)
Oedema peripheral	2 (3.8)
Abdominal discomfort	1 (1.9)
Altered state of consciousness	1 (1.9)
Fall	1 (1.9)
Hepatic function abnormal	1 (1.9)
Liver disorder	1 (1.9)
Loss of consciousness	1 (1.9)
Thirst	1 (1.9)
Serious ADRs	0 (0.0)
Discontinuation due to ADRs	6 (11.5)^b^
Death	0 (0.0)

*ADR,* Adverse drug reaction

^a^Coded using the Japanese version of the Medical Dictionary for Regulatory Activities, version 25.0

^b^A total of 7 ADRs in 6 patients

## Discussion

CIPN includes both painful and non-painful symptoms, both of which can lead to dose reduction or discontinuation of chemotherapy [28]. This interventional part of the MiroCIP study firstly showed the efficacy and safety of mirogabalin in patients with moderate to severe CIPN, including painful symptoms, during chemotherapy. By the end of the first 4 weeks, patients receiving mirogabalin had a significant reduction in NRS scores for both pain and tingling, and this analgesic effect was greater in patients with more severe pain. During 12 weeks of mirogabalin treatment, the incidence of dose reduction, suspension, and discontinuation of chemotherapy due to CIPN remained low. Moreover, there was no worsening of subjective CIPN severity. Mirogabalin was generally well tolerated, and no novel safety concerns arose.

### Pain and tingling

In the present study, NRS score decreased by − 1.5 (27.3%) by week 4 and remained stable at the end of the treatment period (− 1.7, 30.9% reduction at week 12 LOCF). Patients with a baseline NRS of ≥ 6 experienced a − 2.9 reduction (38.7%) in NRS score. Our results are similar to the effects of duloxetine observed in a double-blind placebo-controlled crossover phase 3 study, in which the changes in NRS score were − 1.06 in the duloxetine group and − 0.34 in the placebo group after the initial 5 weeks of treatment [29]. In the randomized controlled trial of duloxetine, patients had completed the CIPN treatment and had at least 3 months of follow-up (i.e. they were patients with persistent symptoms after treatment). Therefore, the study populations of the present study and the randomized controlled trial of duloxetine were different. It should be noted that mirogabalin elicited a significant reduction in NRS scores for both pain and tingling even in patients undergoing chemotherapy.

The Initiative on Methods, Measurement, and Pain Assessment in Clinical Trials (IMMPACT) recommendations state that analgesia can be considered clinical meaningful if it reduces pain severity by ≥ 30% (moderately important improvement) [30]. Although the present study is an open-label single-arm trial, it demonstrated the certain clinical efficacy of mirogabalin for the treatment of CIPN. Furthermore, even patients with severe pain may benefit from the use of mirogabalin as monotherapy.

Tingling is one of the most frequent symptoms in patients with CIPN, but in clinical practice it is often difficult to distinguish between pain and tingling from the patient’s subjective complaints. Previous studies showed that mirogabalin may relieve tingling as well as pain in diabetic peripheral neuropathy [31] and lumbar spinal stenosis [32]. Mirogabalin may also be effective for tingling in CIPN, and it may reduce physicians’ burden in the clinical setting of management of CIPN.

Patients may experience CIPN concurrently with pain related to cancer and/or treatment other than chemotherapy. It is likely difficult for patients to distinguish between CIPN and other causes of pain, and this is a common concern in real-world clinical practice. However, given the pharmacological mechanism of mirogabalin, its analgesic effect in the present study was probably against neuropathic pain. Evidence for this is provided by the consistency between changes in NRS scores for pain (including tingling) and changes in NRS scores for tingling.

### Completion of chemotherapy

CIPN is often the main reason for dose modulation or discontinuation of chemotherapy, both of which can negatively affect patient outcomes [33, 34]. Previous studies showed that the incidence of dose reduction, suspension, and discontinuation of chemotherapy due to CIPN was, respectively, 8%–36%, 9%, and 4.3% for taxane, and 15%, 2%, and 13%–30.8% for oxaliplatin [34–40]. Compared with those previous studies, the results of this study seem to be lower (dose reduction, 5.8%; suspension, 1.9%; discontinuation of chemotherapy, 3.8%). Both symptomatic treatment of pain with CIPN and management of the causative agent are common challenges in clinical practice during chemotherapy. In the present study, the analgesic effect of mirogabalin may have contributed to the completion of chemotherapy in patients with CIPN, suggesting that symptomatic treatment of pain have potential for supporting completion of chemotherapy.

### CIPN severity

Use of symptomatic pain relief medications during chemotherapy may contribute to worsening of CIPN severity, because suppression of subjective symptoms may interfere with appropriate decisions on when to reduce or discontinue chemotherapy. To assess this risk in the present study, the severity of peripheral neuropathy other than pain was evaluated using the Modified TNSr, which is a physician’s objective assessment tool. There was no worsening in the severity of sensory and motor neuropathy assessed by Modified TNSr during the 12 weeks of mirogabalin treatment. We believe that this study provides some objective assessment of the risk that intervention with mirogabalin interferes with the appropriate management of chemotherapy.

### Safety

Our results indicate that mirogabalin is well tolerated in patients with cancer during chemotherapy. Most ADRs in the present study were mild or moderate, and predominantly somnolence, oedema peripheral, and dizziness. These ADRs are similar to those reported for previous studies of mirogabalin and pregabalin [16, 17, 21, 28, 41] and therefore do not represent new safety concerns. The four serious TEAEs that occurred, including one death, were attributed to COVID-19 or cancer progression.

### Limitations

There are some limitations in the present study. First, the study was an open-label, single-arm study without a comparator group; a placebo group could not be used due to ethical concerns. Therefore, the results cannot be definitively attributed to mirogabalin. However, the degree of pain improvement was similar to that found for duloxetine in previous controlled clinical studies [29]. Additionally, to address this limitation, we used several assessment tools including the NRS and CTCAE, which are commonly used in the evaluation of neuropathic pain and CIPN, as well as several patient-reported outcomes, such as FACT/GOG-NTX, PGIC, and EQ-5D-5L. These indicators showed similar trends, suggesting that a certain level of pain relief was due to the effect of mirogabalin.

Second, in the present study, patients were evaluated mostly by oncologists who did not routinely carry out neurological examinations such as testing of tendon reflexes. Physicians were provided with training by viewing a neurologist’s instructional video prior to the start of the study. Previous clinical trials of CIPN often used patients’ subjective evaluations; however, physicians’ objective neurological evaluations are considered essential for accurate evaluation of CIPN severity. The present study design was established via collaboration between oncologists and neurologists and included standardization of assessment procedures, and in this regard may be helpful for future clinical trials of treatments for CIPN.

Third, this was a short-term study (12 weeks). Because multiple cancer types and regimens were involved, a longer study period was not possible. However, chemotherapy with oxaliplatin or taxane is often completed in about 6 months [42]. Considering the time from the start of chemotherapy to the development of moderate to severe CIPN, we believe that the 12-weeks study period may approximately cover the time of interest for CIPN management.

Fourth, no data were collected on modifications of chemotherapy regimens, such as dose reduction from pre-enrollment to enrollment (baseline) or at enrollment. Chemotherapy dose reduction may have had a significant impact on pain and tingling, and on the efficacy results of mirogabalin (its analgesic effects).

## Conclusions

The MiroCIP interventional study shows that mirogabalin has an acceptable safety profile and is effective for pain and tingling due to CIPN in patients with colorectal, gastric, non-small-cell lung, and breast cancers who receives oxaliplatin- and taxane-based chemotherapy. The findings of this study suggest that mirogabalin may be useful for the continuation of chemotherapy and contribute to the management of patients with CIPN undergoing neurotoxic chemotherapy, thereby improving patient QOL and potentially patient outcomes.

### Supplementary Information

**Additional file 1:** **Supplementary Table 1.** Participating institutions and principal investigators for the MiroCIP interventional study. **Supplementary Methods.** **Supplementary Table 2.** Baseline clinical characteristics of patients in the full analysis set (*n *= 52) who had experienced peripheral neuropathic pain. **Supplementary Table 3.** Baseline demographic and clinical characteristics of patients with chemotherapy-induced peripheral neuropathy in the MiroCIP interventional study (per protocol set). **Supplementary Table 4.** Changes in numeric rating scale scores for pain, including tingling (the primary endpoint) and for tingling alone and sleep disturbance (secondary endpoints) from baseline to week 12 in patients with chemotherapy-induced peripheral neuropathy (full analysis set and per protocol set). **Supplementary Table 5.** Incidence of dose reduction or discontinuation of chemotherapya (per protocol set, *n *= 30). **Supplementary Table 6.** Numbers of patients with change in grade of chemotherapy-induced peripheral neuropathy according to the Common Terminology Criteria for Adverse Events (full analysis set and per protocol set). **Supplementary Table 7.** Changes in Functional Assessment of Cancer Therapy/Gynecologic Oncology Group Neurotoxicity subscale (FACT/GOG-NTX) and Modified Total Neuropathy Score-Reduced (TNSr) total scores from baseline to week 12 in patients with chemotherapy-induced peripheral neuropathy (full analysis set and per protocol set). **Supplementary Table 8.** EuroQoL five-dimensional descriptive system index value from baseline to week 12, and Patient Global Impression of Change score at week 12, in patients with chemotherapy-induced peripheral neuropathy (full analysis set and per protocol set). **Supplementary Table 9.** Incidences of treatment-emergent adverse events in patients with chemo.therapy-induced peripheral neuropathy in the MiroCIP interventional study (safety analysis set).

## Data Availability

The datasets generated during and/or analyzed during the current study are not publicly available but are available from the corresponding author and Daiichi Sankyo Co., Ltd., a study sponsor, on reasonable request.

## Abbreviations

ADR: Adverse drug reaction
ASCO: American Society of Clinical Oncology
CI: Confidence interval
CIPN: Chemotherapy-induced peripheral neuropathy
CTCAE: Common Terminology Criteria for Adverse Events
EQ-5D-5L: EuroQol five-dimensional descriptive system
FACT/GOG-NTX: Functional Assessment of Cancer Therapy/Gynecologic Oncology Group Neurotoxicity subscale
FAS: Full analysis set
IMMPACT: Initiative on Methods, Measurement, and Pain Assessment in Clinical Trials
LOCF: Last observation carried forward
Modified TNSr: Modified Total Neuropathy Score-Reduced
NRS: Numeric rating scale
NSAID: Non-steroidal anti-inflammatory drug
PGIC: Patient Global Impression of Change
PPS: Per protocol set
SD: Standard deviation
TEAE: Treatment-emergent adverse event
