# Supplementary material for: Efficacy and safety of mirogabalin for chemotherapy-induced peripheral neuropathy: a prospective single-arm trial (MiroCIP study)
Source: BMC Cancer. 2023 Nov 11;23:1098. doi: 10.1186/s12885-023-11560-4 (PMC10640752; doi:10.1186/s12885-023-11560-4)
Supplement: Supplementary file 1 — Additional file 1: Supplementary Table 1. Participating institutions and principal investigators for the MiroCIP interventional study. Supplementary Methods. Supplementary Table 2. Baseline clinical characteristics of patients in the full analysis set (n = 52) who had experienced peripheral neuropathic pain. Supplementary Table 3. Baseline demographic and clinical characteristics of patients with chemotherapy-induced peripheral neuropathy in the MiroCIP interventional study (per protocol set). Supplementary Table 4. Changes in numeric rating scale scores for pain, including tingling (the primary endpoint) and for tingling alone and sleep disturbance (secondary endpoints) from baseline to week 12 in patients with chemotherapy-induced peripheral neuropathy (full analysis set and per protocol set). Supplementary Table 5. Incidence of dose reduction or discontinuation of chemotherapya (per protocol set, n = 30). Supplementary Table 6. Numbers of patients with change in grade of chemotherapy-induced peripheral neuropathy according to the Common Terminology Criteria for Adverse Events (full analysis set and per protocol set). Supplementary Table 7. Changes in Functional Assessment of Cancer Therapy/Gynecologic Oncology Group Neurotoxicity subscale (FACT/GOG-NTX) and Modified Total Neuropathy Score-Reduced (TNSr) total scores from baseline to week 12 in patients with chemotherapy-induced peripheral neuropathy (full analysis set and per protocol set). Supplementary Table 8. EuroQoL five-dimensional descriptive system index value from baseline to week 12, and Patient Global Impression of Change score at week 12, in patients with chemotherapy-induced peripheral neuropathy (full analysis set and per protocol set). Supplementary Table 9. Incidences of treatment-emergent adverse events in patients with chemo.therapy-induced peripheral neuropathy in the MiroCIP interventional study (safety analysis set). [file 12885_2023_11560_MOESM1_ESM.docx]

**Additional file 1: Supplementary files**

**Supplementary Table 1** Participating institutions and principal investigators for the MiroCIP interventional study

| **Institution** | **Principal investigator at study site** |
| --- | --- |
| Chiba University Hospital | Satoshi Kuwabara^a^ |
| Kimitsu Chuo Hospital | Norio Ureshino |
| National Cancer Center Hospital East | Yoichi Naito |
| Chiba Cancer Center | Tadamichi Denda |
| National Hospital Organization Chiba Medical Center | Daisuke Satomi |
| Toranomon Hospital | Yuko Tanabe |
| Chiba Rosai Hospital | Takeshi Suzuki |
| National Hospital Organization Takasaki General Medical Center | Atsushi Naganuma |
| Gunma Prefectural Cancer Center | Yasuhiro Yanagita |
| Saitama City Hospital | Katsutoshi Sekine |
| Tsuchiura Kyodo General Hospital | Fumihiko Kusano |
| JISENKAI Healthcare Incorporated Foundation, Aizawa Hospital | Masato Nakamura |

^a^ Study principal investigator.

**Supplementary Methods**

**Full eligibility criteria**

Of patients enrolled in the registrational study or from outside the registrational study, patients who provided the written informed consent to participate in and met the eligibility criteria for this interventional study were enrolled.

Included in the MiroCIP registrational study were patients (aged ≥ 20 years) with the following cancers and due to start treatment with the following chemotherapy regimens within 14 days of enrollment: colorectal cancer with a regimen including oxaliplatin; gastric cancer with a regimen including oxaliplatin or a taxane; and non-small-cell lung or breast cancers with a regimen including a taxane. These patients, in addition to patients (also aged ≥ 20 years) with the same four cancers and undergoing oxaliplatin- or taxane-containing chemotherapy but recruited from outside the registrational study, were eligible to participate in the MiroCIP interventional study if they had chemotherapy-induced peripheral sensory neuropathy of grade ≥ 2 according to the Common Terminology Criteria for Adverse Events (CTCAE) version 5.0 [1] and a numeric rating scale (NRS) score of ≥ 4 for pain, including tingling. This approach to recruitment, with eligibility open both to patients already enrolled in the MiroCIP registry and to patients meeting similar inclusion criteria (Fig. 1), was used to ensure that the prespecified target sample size was reached.

Excluded from the registrational study were patients with severe diabetes mellitus (HbA1c [NGSP] > 8.4%); with a history of heavy alcohol consumption or alcohol dependence; with severe cervical or lumbar spondylosis; with poor life expectancy; or with pain due to other diseases or factors that would adversely affect the assessment of CIPN pain; in addition to those for whom participation in research was judged inappropriate by an investigator.

Excluded from the interventional study were patients with serious renal, cardiac, or hepatic disease; with creatinine clearance < 30 mL/min (Cockcroft–Gault formula) in the 3 months before enrollment; with a history of hypersensitivity to the components of the mirogabalin tablets; being scheduled for chemotherapy modification or surgery within 3 months of enrollment; or being judged by investigators to be otherwise unsuitable as study participants. Patients who were pregnant, possibly pregnant, or lactating were also excluded.

**Reference**

1. National Cancer Institute, Cancer Therapy Evaluation Program. National Cancer Institute. <https://ctep.cancer.gov/protocolDevelopment/electronic_applications/ctc.htm#ctc_50>.

**Causal relationships with the study drug**

In the protocol, the definition of a causal relationship with the study drug was as follows, and each attending physician determined the presence or absence of a causal relationship based on this definition.

Definition for *Related to the study drug*

1. There is a reasonable temporal correlation between the occurrence of the adverse event and the administration of the investigational drug, and it is not reasonable to attribute the adverse event to the subject‘s condition or to factors other than the investigational drug (e.g. primary disease, complications, concomitant medications) and an association with the investigational drug cannot be ruled out.

2. There is a reasonable temporal correlation between the occurrence of the adverse event and the administration of the investigational drug, which can be explained as a known reaction to or pharmacological effect of the investigational drug or a similar compound.

Definition for *Unrelated to the study drug*

1. If there is no reasonable temporal correlation between the occurrence of the adverse event and the administration of the investigational drug, or if it is reasonable to conclude that the adverse event was caused by factors other than the subject’s condition or the investigational drug (e.g. primary illness, complications, concomitant medications) and that an association with the investigational drug can be ruled out.

2. If there is no treatment with mirogabalin and the adverse event occurs.

**Supplementary Table 2** Baseline clinical characteristics of patients in the full analysis set (*n* = 52) who had experienced peripheral neuropathic pain

| **Characteristic** | ***n* (%)** |
| --- | --- |
| History of peripheral neuropathic pain | 5 (9.6) |
| Cervical or lumbar spondylosis | 2 (3.8) |
| Postoperative neuropathy | 1 (1.9) |
| Other peripheral neuropathy | 2 (3.8) |

**Supplementary Table 3** Baseline demographic and clinical characteristics of patients with chemotherapy-induced peripheral neuropathy in the MiroCIP interventional study (per protocol set)

| **Characteristic** | **Per protocol set (*n* = 30)^a^** |
| --- | --- |
| Age, years | 64.5 ± 9.7 |
| Sex, male/female | 18 (60.0) / 12 (40.0) |
| Body mass index, kg/m^2^ | 22.43 ± 2.68 |
| Creatinine clearance, mL/min | 86.38 ± 25.11 |
| ≥ 60 | 26 (86.7) |
| 30 to < 60 | 4 (13.3) |
| Smoking status, current/ previous/ never | 4 (13.3) / 15 (50.0) / 11 (36.7) |
| Alcohol consumption habit | 20 (66.7) |
| Cancer type, C / G / N / B | 20 (66.7) / 2 (6.7) / 3 (10.0) / 5 (16.7) |
| Cancer stage, III / IV / R | 5 (16.7) / 12 (40.0) / 13 (43.3) |
| Performance status, 0 / 1 | 22 (73.3), 8 (26.7) |
| Timing of chemotherapy, post / non-peri | 4 (13.3) / 26 (86.7) |
| History of radiotherapy | 6 (20.0) |
| Chemotherapeutic agent included in regimen, O / T^b^ | 20 (66.7) / 10 (33.3) |
| Accumulated dose per body surface area, mg/m^2^, O/T^c^ | 1322.27±549.14 / 1573.37±2117.05 |
| History of peripheral neuropathic pain^d^ | 1 (3.3) |
| History of neurotoxic chemotherapy | 7 (23.3) |
| Oxaliplatin | 5 (16.7) |
| Carboplatin | 1 (3.3) |
| Cisplatin | 1 (3.3) |
| Docetaxel | 1 (3.3) |

*B*, breast cancer; *C*, colorectal cancer; *G*, gastric cancer; *non-peri*, non-perioperative; *N*, non-small-cell lung cancer; *O*, oxaliplatin; *post*, postoperative; *R*, recurrence after surgery; *SD*, standard deviation; *T*, taxane.

^a^ Mean ± SD or *n* (%).

^b^ Taxane included docetaxel (*n* =1, 3.3%) and paclitaxel (*n* = 9, 30.0%).

^c^ These data were at the end of the study.

^d^ Postoperative neuropathy.

**Supplementary Table 4** Changes in numeric rating scale scores for pain, including tingling (the primary endpoint) and for tingling alone and sleep disturbance (secondary endpoints) from baseline to week 12 in patients with chemotherapy-induced peripheral neuropathy (full analysis set and per protocol set)

| **NRS score** | **Full analysis set** | | | | **Per protocol set** | | | |
| --- | --- | --- | --- | --- | --- | --- | --- | --- |
|  | **Baseline  (*n* = 52)** | **Week 4  (*n* = 50)** | **Week 12  (*n* = 41)** | **LOCF  (*n* = 51)** | **Baseline  (*n* = 30)** | **Week 4  (*n* = 29)** | **Week 12  (*n* = 24)** | **LOCF  (*n* = 30)** |
| Pain (including tingling) |  |  |  |  |  |  |  |  |
| Mean ± SD | 5.5 ± 1.5 | 4.1 ± 2.0 | 4.0 ± 2.2 | 3.8 ± 2.2 | 5.3 ± 1.2 | 4.0 ± 1.9 | 3.7 ± 2.1 | 3.8 ± 2.0 |
| Change from baseline | NA | −1.5 ± 2.3 | −1.5 ± 2.3 | −1.7 ± 2.5 | NA | −1.2 ± 2.0 | −1.5 ± 1.6 | −1.5 ± 1.7 |
| 95% CI | NA | −2.1 to −0.8 | −2.3 to −0.8 | −2.4 to −1.0 | NA | −2.0 to −0.5 | −2.2 to −0.8 | −2.1 to −0.8 |
| *p* value vs baseline^a^ | NA | < 0.001 | < 0.001 | < 0.001 | NA | 0.002 | < 0.001 | < 0.001 |
| Tingling alone |  |  |  |  |  |  |  |  |
| Mean ± SD | 5.3 ± 1.6 | 4.0 ± 1.9 | 4.1 ± 2.2 | NA | 5.0 ± 1.6 | 3.9 ± 1.7 | 3.9 ± 2.0 | NA |
| Change from baseline | NA | −1.4 ± 2.2 | −1.2 ± 2.4 | NA | NA | −1.3 ± 1.7 | −1.1 ± 2.0 | NA |
| 95% CI | NA | −2.0 to −0.8 | −1.9 to −0.4 | NA | NA | −1.9 to −0.6 | −2.0 to −0.3 | NA |
| *p* value vs baseline^a^ | NA | < 0.001 | 0.003 | NA | NA | < 0.001 | 0.010 | NA |
| Sleep disturbance |  |  |  |  |  |  |  |  |
| Mean ± SD | 1.1 ± 1.7 | 0.7 ± 1.7 | 0.5 ± 1.7 | NA | 1.1 ± 1.7 | 0.8 ± 1.5 | 0.4 ± 1.6 | NA |
| Change from baseline | NA | −0.3 ± 2.1 | −0.2 ± 2.0 | NA | NA | −0.4 ± 2.0 | −0.4 ± 2.2 | NA |
| 95% CI | NA | −0.9 to 0.3 | −0.8 to 0.4 | NA | NA | −1.1 to 0.4 | −1.3 to 0.6 | NA |
| *p* value vs baseline^a^ | NA | 0.292 | 0.534 | NA | NA | 0.318 | 0.413 | NA |

*CI*, confidence interval; *LOCF*, last observation carried forward; *NA*, not applicable; NRS, numeric rating scale; *SD*, standard deviation.

^a^ Determined by paired *t*-test.

**Supplementary Table 5** Incidence of dose reduction or discontinuation of chemotherapy^a^ (per protocol set, *n* = 30)

| **Event** | ***n* (%)** |
| --- | --- |
| **Due to any cause** |  |
| No dose reduction, suspension, or discontinuation | 11 (36.7) |
| Dose reduction | 3 (10.0) |
| Suspension | 7 (23.3) |
| Discontinuation | 10 (33.3) |
| **Due to CIPN** |  |
| No dose reduction, suspension, or discontinuation | 24 (80.0) |
| Dose reduction | 3 (10.0) |
| Suspension | 1 (3.3) |
| Discontinuation | 2 (6.7) |

*CIPN*, chemotherapy-induced peripheral neuropathy.

^a^ Including oxaliplatin or a taxane.

**Supplementary Table 6** Numbers of patients with change in grade of chemotherapy-induced peripheral neuropathy according to the Common Terminology Criteria for Adverse Events (full analysis set and per protocol set)

| **Full analysis set^a^** | | | | | | | | | | |
| --- | --- | --- | --- | --- | --- | --- | --- | --- | --- | --- |
| **Baseline** | **Week 4 (*n* = 52)** | | | | | **Week 12 (*n* = 52)** | | | | |
|  | **No CIPN** | **Grade 1** | **Grade 2** | **Grade 3** | **Grade 4** | **No CIPN** | **Grade 1** | **Grade 2** | **Grade 3** | **Grade 4** |
| No CIPN | 0 (0.0) | 0 (0.0) | 0 (0.0) | 0 (0.0) | 0 (0.0) | 0 (0.0) | 0 (0.0) | 0 (0.0) | 0 (0.0) | 0 (0.0) |
| Grade 1 | 0 (0.0) | 0 (0.0) | 0 (0.0) | 0 (0.0) | 0 (0.0) | 0 (0.0) | 0 (0.0) | 0 (0.0) | 0 (0.0) | 0 (0.0) |
| Grade 2 | 2 (3.8) | 6 (11.5) | 36 (69.2) | 2 (3.8) | 0 (0.0) | 1 (1.9) | 3 (5.8) | 35 (67.3) | 0 (0.0) | 0 (0.0) |
| Grade 3 | 0 (0.0) | 0 (0.0) | 3 (5.8) | 1 (1.9) | 0 (0.0) | 0 (0.0) | 0 (0.0) | 2 (3.8) | 0 (0.0) | 0 (0.0) |
| Grade 4 | 0 (0.0) | 0 (0.0) | 0 (0.0) | 0 (0.0) | 0 (0.0) | 0 (0.0) | 0 (0.0) | 0 (0.0) | 0 (0.0) | 0 (0.0) |
| **Per protocol set^b^** | | | | | | | | | | |
| **Baseline** | **Week 4 (*n* = 30)** | | | | | **Week 12 (*n* = 30)** | | | | |
|  | **No CIPN** | **Grade 1** | **Grade 2** | **Grade 3** | **Grade 4** | **No CIPN** | **Grade 1** | **Grade 2** | **Grade 3** | **Grade 4** |
| No CIPN | 0 (0.0) | 0 (0.0) | 0 (0.0) | 0 (0.0) | 0 (0.0) | 0 (0.0) | 0 (0.0) | 0 (0.0) | 0 (0.0) | 0 (0.0) |
| Grade 1 | 0 (0.0) | 0 (0.0) | 0 (0.0) | 0 (0.0) | 0 (0.0) | 0 (0.0) | 0 (0.0) | 0 (0.0) | 0 (0.0) | 0 (0.0) |
| Grade 2 | 1 (3.3) | 3 (10.0) | 23 (76.7) | 0 (0.0) | 0 (0.0) | 0 (0.0) | 3 (10.0) | 21 (70.0) | 0 (0.0) | 0 (0.0) |
| Grade 3 | 0 (0.0) | 0 (0.0) | 1 (3.3) | 1 (3.3) | 0 (0.0) | 0 (0.0) | 0 (0.0) | 0 (0.0) | 0 (0.0) | 0 (0.0) |
| Grade 4 | 0 (0.0) | 0 (0.0) | 0 (0.0) | 0 (0.0) | 0 (0.0) | 0 (0.0) | 0 (0.0) | 0 (0.0) | 0 (0.0) | 0 (0.0) |

*CIPN*, chemotherapy-induced peripheral neuropathy.

^a^ In the full analysis set, data were missing for 2 and 11 patients at weeks 4 and 12, respectively.

^b^ In the per protocol set, data were missing for 1 patient and 6 patients at weeks 4 and 12, respectively.

**Supplementary Table 7** Changes in Functional Assessment of Cancer Therapy/Gynecologic Oncology Group Neurotoxicity subscale (FACT/GOG-NTX) and Modified Total Neuropathy Score-Reduced (TNSr) total scores from baseline to week 12 in patients with chemotherapy-induced peripheral neuropathy (full analysis set and per protocol set)

|  | **Full analysis set** | | | **Per protocol set** | | |
| --- | --- | --- | --- | --- | --- | --- |
| **Score** | **Baseline** | **Week 4** | **Week 12** | **Baseline** | **Week 4** | **Week 12** |
| FACT/GOG-NTX total score, *n* | 51 | 49 | 41 | 30 | 28 | 24 |
| Mean ± SD | 12.7 ± 6.9 | 10.1 ± 6.8 | 10.8 ± 7.4 | 11.0 ± 6.4 | 10.3 ± 7.1 | 11.1 ± 7.2 |
| Change from baseline | NA | −2.6 ± 6.2 | −1.6 ± 6.4 | NA | −0.6 ± 5.6 | 0.2 ± 6.7 |
| 95% CI | NA | −4.4 to −0.8 | −3.6 to 0.4 | NA | −2.8 to 1.5 | −2.6 to 3.0 |
| *p* value vs baseline^a^ | NA | 0.006 | 0.114 | NA | 0.551 | 0.903 |
| Modified TNSr total score, *n* | 52 | NA | 41 | 30 | NA | 24 |
| Mean ± SD | 9.3 ± 3.1 | NA | 9.1 ± 2.5 | 8.6 ± 2.8 | NA | 8.9 ± 2.6 |
| Change from baseline | NA | NA | −0.5 ± 3.2 | NA | NA | 0.2 ± 2.6 |
| 95% CI | NA | NA | −1.5 to 0.5 | NA | NA | −0.9 to 1.3 |
| *p* value vs baseline^a^ | NA | NA | 0.284 | NA | NA | 0.758 |

*CI*, confidence interval; *FACT/GOG-NTX*, Functional Assessment of Cancer Therapy/Gynecologic Oncology Group Neurotoxicity subscale; *Modified TNSr*, Modified Total Neuropathy Score-Reduced; *NA*, not applicable; *SD*, standard deviation.

^a^ Determined by paired *t*-test.

**Supplementary Table 8** EuroQoL five-dimensional descriptive system index value from baseline to week 12, and Patient Global Impression of Change score at week 12, in patients with chemotherapy-induced peripheral neuropathy (full analysis set and per protocol set)

| **Score** | **Full analysis set** | **Per protocol set^a^** |
| --- | --- | --- |
| EQ-5D-5L index value at baseline, *n* | 52 | 30 |
| Mean ± SD | 0.7824 ± 0.1373 | 0.8071 ± 0.1149 |
| Median (minimum, maximum) | 0.8116 (0.246, 1.000) | 0.8228 (0.526, 1.000) |
| EQ-5D-5L index value at week 12, *n* | 40 | 24 |
| Mean ± SD | 0.7910 ± 0.1828 | 0.7743 ± 0.1982 |
| Median (minimum, maximum) | 0.8286 (0.099, 1.000) | 0.8246 (0.099, 1.000) |
| Change from baseline | 0.0128 ± 0.1672 | −0.0312 ± 0.1828 |
| 95% CI | −0.0406 to 0.0663 | −0.1084 to 0.0460 |
| *p* value vs baseline^b^ | 0.630 | 0.412 |
| PGIC score at week 12, *n* | 33 | 20 |
| 1 (‘very much improved’) | 0 (0.0) | 0 (0.0) |
| 2 (‘much improved’) | 8 (24.2) | 4 (20.0) |
| 3 (‘minimally improved’) | 16 (48.5) | 10 (50.0) |
| 4 (‘no change’) | 6 (18.2) | 4 (20.0) |
| 5 (‘minimally worse’) | 3 (9.1) | 2 (10.0) |
| 6 (‘much worse’) | 0 (0.0) | 0 (0.0) |
| 7 (‘very much worse’) | 0 (0.0) | 0 (0.0) |
| PGIC (score ≤ 2) | 8 (24.2) | 4 (20.0) |
| PGIC (score ≤ 3) | 24 (72.7) | 14 (70.0) |

*CI*, confidence interval; *EQ-5D-5L*, EuroQol five-dimensional descriptive system; *PGIC*, Patient Global Impression of Change; *SD*, standard deviation.

^a^ *n* (%) unless otherwise specified.

^b^ Determined by paired *t*-test.

**Supplementary Table 9** Incidences of treatment-emergent adverse events in patients with chemotherapy-induced peripheral neuropathy in the MiroCIP interventional study (safety analysis set)

| **TEAE^a^** | ***n* (%)** |
| --- | --- |
| Overall | 40 (76.9) |
| Occurring in ≥ 2 patients |  |
| Somnolence | 7 (13.5) |
| Oedema peripheral | 6 (11.5) |
| Pyrexia | 6 (11.5) |
| Dizziness | 5 (9.6) |
| Neutrophil count decreased | 5 (9.6) |
| Stomatitis | 4 (7.7) |
| Anaemia | 3 (5.8) |
| Neuropathy peripheral | 3 (5.8) |
| Platelet count decreased | 3 (5.8) |
| Contusion | 2 (3.8) |
| COVID-19 | 2 (3.8) |
| Liver disorder | 2 (3.8) |
| Peripheral sensory neuropathy | 2 (3.8) |
| Pruritus | 2 (3.8) |
| Rash | 2 (3.8) |
| Vomiting | 2 (3.8) |
| Serious TEAEs | 4 (7.7) |
| Discontinuation due to TEAEs | 8 (15.4)^b^ |
| Death | 1 (1.9) |

*TEAEs*, treatment-emergent adverse events.

^a^ Coded using the Japanese version of the Medical Dictionary for Regulatory Activities, version 25.0.

^b^ A total of 9 TEAEs in 8 patients.
